# Uncovering the Mechanism of the Effects of *Pien-Tze-Huang* on Liver Cancer Using Network Pharmacology and Molecular Docking

**DOI:** 10.1155/2020/4863015

**Published:** 2020-09-07

**Authors:** Shanghui Liu, Run Wang, Yan Lou, Jia Liu

**Affiliations:** ^1^School of Fundamental Sciences, China Medical University, Shenyang 110122, Liaoning, China; ^2^Department of Neurosurgery, The 1st Hospital of China Medical University, Shenyang 110001, Liaoning, China

## Abstract

*Pien-Tze-Huang* (PTH) has a long history in the treatment of liver cancer. However, its molecular mechanism of action remains unclear. TCMSP and TCM were used to collect the active ingredients. Bioactive compounds targets were predicted by reverse pharmacophore models. The antiliver cancer targets of PTH were selected by gene comparison of liver cancer in the GEO database. Molecular docking was used to verify the binding activity of the targets and the active ingredients. The DAVID was used to analyze the gene function and signal pathway. A model was built with Cytoscape. The core genes were obtained by PPI network. We screened the 4 main medicinal ingredients of PTH to obtain 16 active ingredient, 190 potential targets, and 6 core genes. We found that active small molecules exert anticancer effects by multiple pathways. The core genes were involved in multiple biological processes. We also found that eight chemical components play a greater role in inhibiting liver cancer. PTH achieves the effect of inhibiting liver cancer through the synergistic effect of multiple components, multiple targets, and multiple pathways. This study provides a potential scientific basis for further elucidating the molecular mechanism of action of PTH against liver cancer.

## 1. Introduction

Liver cancer is a common clinical malignant tumor of liver. The incidence ranks the 5th in the world [1]. The fatality rate ranks the 2nd in the world [2]. Currently surgical resection is the main treatment. This is because most of the patients are already in the late stage once diagnosed, so that they can only be treated by drug instead of surgery. Clinically, first-line treatment drugs such as sorafenib are mainly used, but long-term use makes it easy for patients to have tolerance and a variety of adverse reactions [3, 4]. Therefore, exploring effective drugs to prevent and treat liver cancer is becoming an urgent issue. Many clinical studies have proved that Chinese medicine has good effects in improving the symptoms of patients with advanced liver cancer [5], reducing tumor recurrence [6], and controlling disease progression [7].

*Pien-Tze-Huang* (PTH), a classic Chinese medicine prescription, is made from Chinese ingredients such as *Rhizoma Notoginseng* (*Sanqi* in Chinese), *Moschus* (*Shexiang* in Chinese), *Calculus Bovis* (*Niuhuang* in Chinese), and *Snake Gall* (*Shedan* in Chinese) [8]. It is a proprietary Chinese medicine with the function of detoxification, anti-inflammation, and immune regulation [9, 10]. PTH has a history of more than 500 years and has been used as a folk medicine in China and Southeast Asia to treat various inflammation-related diseases such as hepatitis. In China, PTH is clinically used to protect the liver [11, 12], treat hepatitis [13], improve liver fibrosis [14], and treat liver cancer [15–17]. Studies on the effects of PTH on lung cancer [18], intestinal cancer [19, 20], osteosarcoma [21], and liver cancer [22] have been reported, but the active ingredients of PTH on liver cancer and the relevant molecular mechanisms are still unclear.

Network pharmacology was developed on the basis of the rapid development of system biology, revealing the complex network relationship between drug-gene-target-disease, systematically observing the intervention and influence of drugs on the disease network, and revealing the molecular mechanism of drug therapy [23, 24]. Traditional Chinese medicine compounds are difficult to study because of their multiple herbs, complex components, and unclear mechanism of action. Network pharmacology provides a new way of thinking for the study of complex traditional Chinese medicine systems [25]. Molecular docking technology is to place small drug molecules (ligands) in the binding area of macromolecular targets (receptors) through computer simulation, predict the binding energy (binding affinity) and binding mode (conformation) of the two by calculating physical and chemical parameters, and then find the lowest energy conformation when the ligands and receptors combine in their active areas [26]. Low binding energy is the basis of stable binding between molecules. The aim of molecular docking is to find the best binding position when the binding energy is the lowest.

In this paper, with the help of network pharmacology, the bioactive compounds and possible molecular network mechanism of PTH against liver cancer are analyzed from a systematic and molecular level. The inhibition of PTH on liver cancer is verified by microarray data of liver cancer in GEO database and molecular docking technology. At the same time, it provides a method to identify the active molecules and target genes involved in complex diseases.

## 2. Materials and Methods

### 2.1. Screening of Active Ingredients in PTH

By searching TCM Database@Taiwan (http://tcm.cmu.edu.tw/), TCMSP (http://tcmspw.com/tcmsp.php), and related literature, we collected the main chemical components of *Calculus Bovis, Moschus, Rhizoma Notoginseng*, and *Snake Gall* in PTH, respectively. The oral bioavailability (OB ≥ 30%), drug-likeness (DS ≥ 0.18), and clear biological activity report [13, 27] were employed to identify the possible bioactive compounds in PTH. 2D and 3D structures and structural graphics of the above compounds were obtained using the PubChem database (https://pubchem.ncbi.nlm.nih.gov/) and saved in mol2 format.

### 2.2. Prediction of Bioactive Compounds Targets for Liver Cancer

Through PharmMapper database (http://www.lilab-ecust.cn/pharmmapper/), we uploaded the mol2 format file of main active ingredient of PTH and used reverse pharmacophore method to get the information of interaction with compound and target protein. The target protein structures were downloaded and accurate information about proteins was further extracted from UniProt database (https://sparql.uniprot.org/), which is a comprehensive resource of protein sequence and rich functional information resources.

RNA sequencing data of liver cancer were obtained from the GSE76427 microarray in the GEO database (https://www.ncbi.nlm.nih.gov/geo/). The mRNA sequencing data contained 115 liver cancer tissues and 52 normal liver tissues. The data used in this article was from GEO open databases and does not require informed consent. Differential analysis was performed using the limma package in R to obtain DEmRNA with thresholds of |log2 fold change (FC)| > 1 and *P* value < 0.05.

Venn diagrams were used to further calculate the intersection of DEmRNA and the potential target genes, which were considered to be the target genes for predicting the active ingredients of PTH.

### 2.3. Active Ingredient-Target Protein Molecular Docking

To investigate the interaction relationship and mechanisms of action between candidate compounds and targets, we conducted a molecular docking study to explore binding activity of the two. We used the RCSB PDB (http://www.rcsb.org/pdb/home/home.do) database to retrieve and download the 3D structure file of the target protein as the receptor. The ligands were prepared based on ChemDraw Ultra 8.0 software for constructing two-dimensional structures and saved in mol2 format. Both of them were coimported into AutoDock Vina for molecular docking calculation. The docking results use binding energy as a reference to screen for the most active ligand molecules and target genes. At present, there is no unified standard for binding energy screening. Binding energy less than 0 means that ligand and receptor can bind spontaneously. The smaller the binding energy is, the more stable the binding between ligand and receptor is. After consulting the literature, it was considered that PTH had better binding activity with binding energy less than −5.0 kJ·mol^−1^ [28], which was the basis for screening candidate target of active ingredient.

### 2.4. Go and Pathways Analysis of Candidate Targets

Potential targets with binding energies less than 5.0 kJ·mol^−1^ were uploaded to the DAVID database (https://david.ncifcrf.gov/) for Gene Ontology (GO) and pathway enrichment analyses (KEGG). The target gene name list was defined as *Homo sapiens*, and the threshold of significant difference was set at *P* < 0.05. The results were plotted in R language.

### 2.5. Component-Target-Pathway Network Construction

To explore the mechanism of PTH with multiple ingredients, targets, and pathways, active ingredient-target-pathway network was built by Cytoscape 3.7.2, which has good binding power of active ingredient and target. Nodes of different colors indicate molecules, target proteins, and associated annotation pathways, and edges indicate molecule-target protein and target protein-pathway interrelationships.

### 2.6. Construction of PPI Network and Key Subnetwork

Based on target protein of PTH, the String (https://string-db.org/) database was used to construct a protein PPI network. Using Cytoscape 3.7.2 software, we selected core genes through maximal clique centrality (MCC) topological analysis method in cytoHubba and built the key subnetwork of PTH target protein.

### 2.7. Visualization of Docking between Core Genes and Active Ingredients

The target protein represented by the core genes and the active ingredient of interaction were analyzed to find the active ingredient with the minimum binding energy. Molecular visualization software PyMOL can be used to show docking effect conformation.

## 3. Results

### 3.1. Screening Results of Active Ingredients in PTH

By mining TCMSP and TCM, the main chemical components, such as *Calculus Bovis*, *Moschus, Rhizoma Notoginseng*, and *Snake Gall*, in PTH were identified. During the screening process, it was found that some of the components failed to meet the requirements of OB ≥ 30% and DL ≥ 0.18. However, those components in PTH were relatively high and had been proved to have clearly biological activity by literatures; they were also included in the study of active components. At last, 18 PTH active ingredients were collected, including 6 *Rhizoma Notoginseng*, 7 *Calculus Bovis*, 1 *Moschus*, and 4 *Snake galls*. 16 remained after weight removal. Detailed data is listed in Table 1.

### 3.2. Prediction Results of Bioactive Compounds Targets in PTH for Liver Cancer

**Figure 1:**
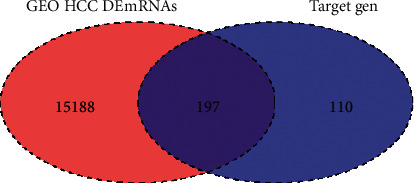
Venn of GEO DEmRNA and target genes.

Through the PharmMapper, 4505 potential targets were obtained by analysing 16 active ingredients in PTH. The target names were input into UniProt database, and all the target names were corrected to the gene names of the target; 986 target genes were obtained. 307 target genes were obtained after further gene deduplication. Then, 307 target genes and 15385 differentially expressed mRNA (DEmRNA) of GEO were intersected, and 197 potential liver cancer related targets were screened by the intersection of Venn (Figure 1).

### 3.3. Molecular Docking Results

**Figure 2:**
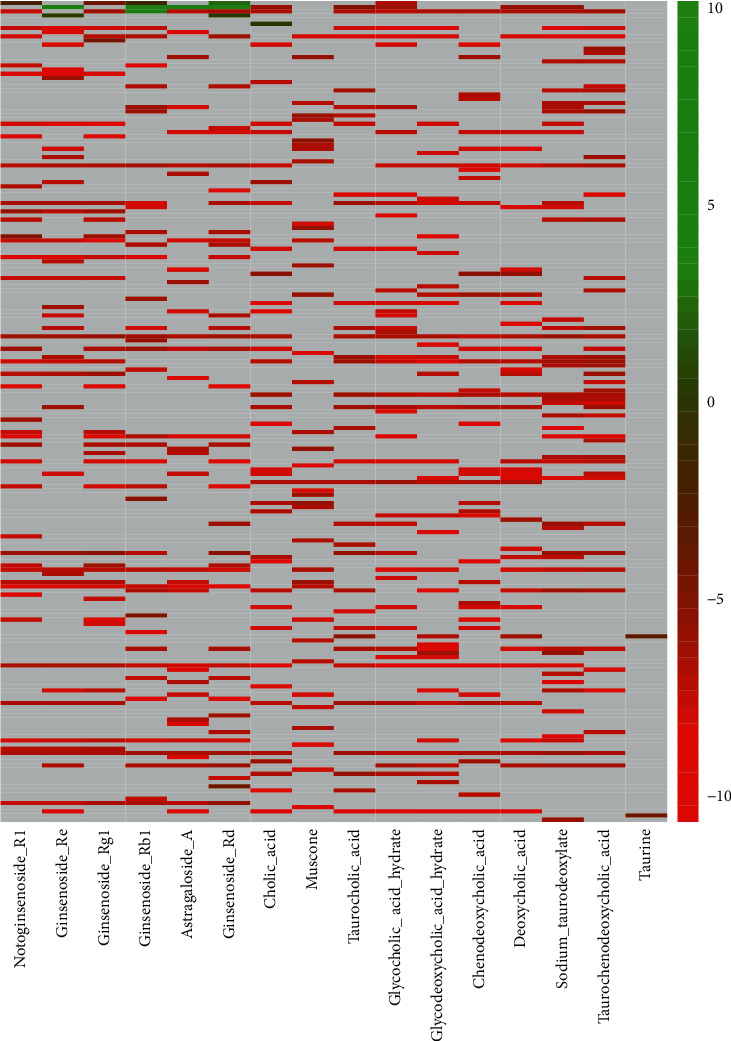
Heatmap plot of molecular docking between active ingredients and target genes. Red represents higher binding activity, green represents lower binding activity levels, and gray represents no connection between target gene and active ingredient.

With 16 active ingredients as ligands and 197 target protein genes as receptors, 614 groups of corresponding relationships were formed. The binding energy of each group can be calculated by molecular docking, and the binding energy of docking results can be displayed by heatmap (Figure 2). Red indicates that the binding energy is negative. Green indicates positive. Gray indicates that protein gene is not the target of active ingredient. Being dominated by red means that most of the active ingredients in PTH had good interaction and binding activity with target. The 190 target genes with better binding activity were obtained by screening out targets with binding energy less than −5.

### 3.4. GO and KEGG Analysis of Target Genes

**Figure 3:**
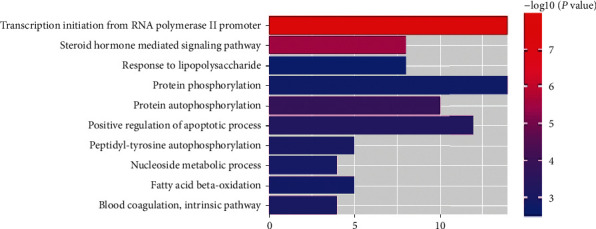
Enriched gene ontology terms for biological processes from active ingredients of PTH.

**Figure 4:**
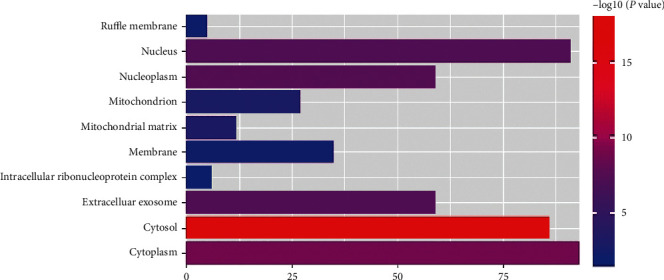
Enriched gene ontology terms for cell component from active ingredients of PTH.

**Figure 5:**
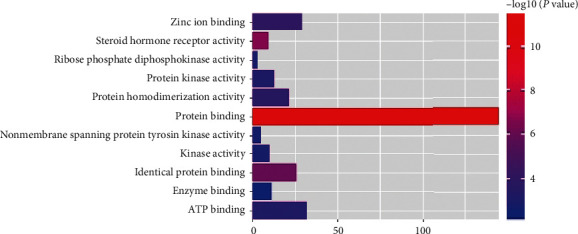
Enriched gene ontology terms for molecular function from active ingredients of PTH.

We used DAVID database to analyze the process of GO biological function and the enrichment of KEGG metabolic pathway of 190 target genes with good binding activity. The threshold was set to *P* < 0.05. There were 71, 45, and 25 enrichment items of biological processes (BP), cell component (CC), and molecular function (MF), respectively. Top 10 records were screened from small to large *P* values. Items related to BP mainly focused on transcription initiation from RNA polymerase II promoter, steroid hormone mediated signaling pathway, protein autophosphorylation, positive regulation of apoptotic process, protein phosphorylation, response to lipopolysaccharide, etc. (Figure 3). The items related to CC mainly involved cytosol, cytoplasm, nucleoplasm, extracellular exosome, nucleus, etc. (Figure 4). MF related items mainly involved protein binding, same protein binding, zinc ion binding, protein homopolymer activity, ATP binding, protein kinase activity, etc. (Figure 5).

**Figure 6:**
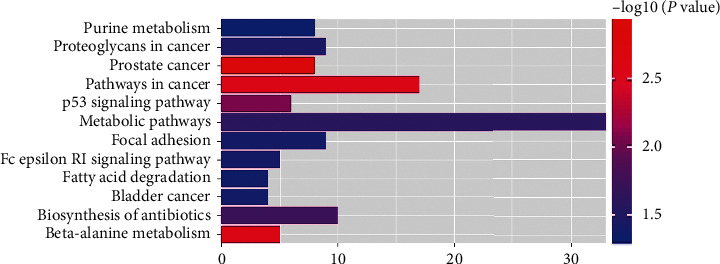
Enriched KEGG pathways of potential targets from active ingredients of PTH.

KEGG signaling pathway analysis is a process in which multiple proteins interact to regulate cell function and metabolism. 12 signal pathways with *P* < 0.05 mainly include metabolic pathways, pathways in cancer, biosynthesis of antibiotics, proteoglycans in cancer, focal adhesion, purine metabolism, etc. (Figure 6).

### 3.5. Construction Results of Component-Target-Pathway Network

**Figure 7:**
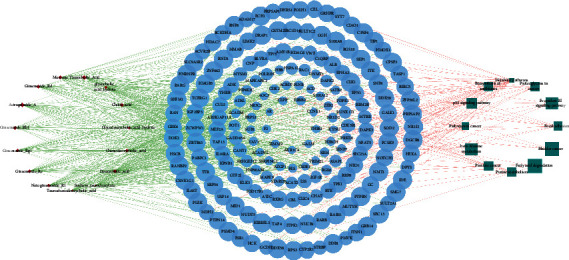
Component-target-pathway network. Red circles represent components, blue ellipses represent targets, and green squares represent pathways.

The component-target-pathway network was constructed by using Cytoscape 3.7.2. Green dot indicated the active component of PTH. Blue was the target protein of the active component, and red was KEGG pathway (Figure 7). It can be seen from the figure that 16 active components of PTH act on 190 targets and distribute in 12 different metabolic pathways. The network degree value and the number of target genes of each active component in PTH were calculated (Table 2). The degree of 15 compounds was more than 30. In particular, the numbers of target genes targeted by sodium taurodeoxylate, taurochenodeoxycholic acid, and glycocholic acid hydrate were 48, 46, and 43. Only the number of target genes targeted by *Taurine* was as few as 2, and the binding energies were −4.3 and −3.2. The target factor less than −5 was 0. PTH has the mechanism of multicomponent, multitarget, and multichannel.

### 3.6. Construction Results of PPI Network and Key Subnetwork

**Figure 8:**
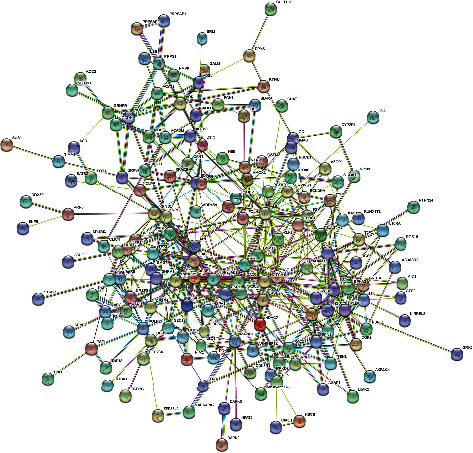
PPI network of target proteins.

**Figure 9:**
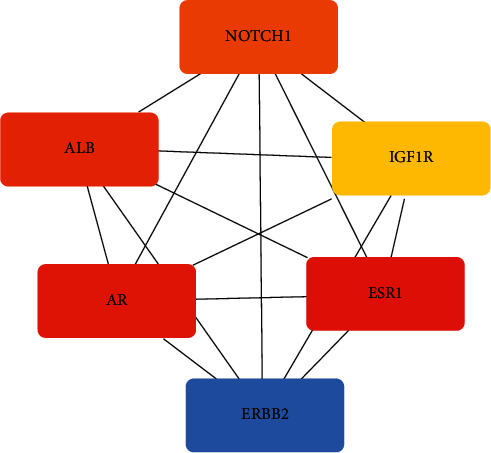
Key subnetwork with core genes.

The PPI network was constructed by analysing 190 target proteins in STRING database. The minimum required interaction score of PPI network is 0.4 by default. The disconnected nodes are hidden. After screening, 167 targets can interact with proteins, 492 edges represent the interaction relationship between proteins and the average degree of each node is 5.9. PPI network of PTH is shown in Figure 8. Using the MCC algorithm of cytoHubba plug-in in Cytoscape, a PPI key subnetwork composed of six genes with the highest score is obtained. The PPI key subnetwork with ESR1, AR, ALB, NOTCH1, ERBB2, and IGF1R has the most connectivity and contains more genes than other subnets, which can avoid missing the key factors related to disease (Figure 9).

### 3.7. Visual Analysis of Docking between Core Genes and Active Ingredients

**Figure 10:**
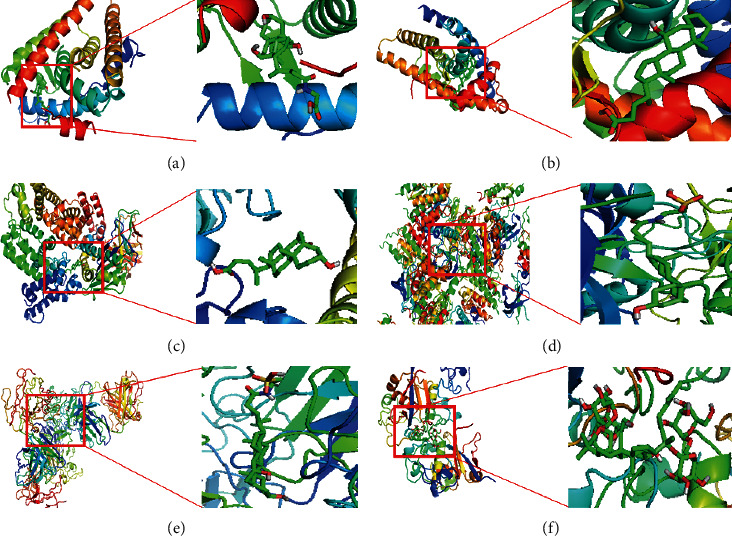
Molecular docking charts of core genes and active ingredients. (a) ESR1 and glycocholic acid hydrate. (b) AR and chenodeoxycholic acid. (c) ALB and chenodeoxycholic acid. (d) IGF1 and sodium taurodeoxylate. (e) ERBB2 and taurochenodeoxycholic acid. (f) NOTCH1 and ginsenoside Rb1.

Molecular docking of the core genes to the active ingredients with high mutual affinity results is shown in Table 3. The corresponding molecular docking effects are shown in Figure 10. It shows the best conformation when the binding energy between the small molecule and the target protein is the lowest and shows the interaction mode between the drug molecule and the core target.

## 4. Discussion

There are 16 main chemical components in PTH, which act on 307 target genes in human body. 197 target genes related to liver cancer were obtained by crossing with hepatoma genes in GEO database. In order to verify the binding activity of chemical components and target genes, 16 chemical components were combined with 197 target genes for molecular docking, and 190 target genes with binding energy less than −5 were finally obtained. Then, GO enrichment analysis, KEGG pathway analysis, PPI network, and key subnet construction as well as the establishment of component-target-pathway network were carried out, and the synergistic mechanism of PTH multicomponent, multitarget, and multipathway was obtained.

PTH has many active target genes against liver cancer. Six core active target genes (ESR1, ERBB2, IGF1R, AR, NOTCH1, and ALB) were obtained by constructing PPI subnetwork. The antihepatocellular carcinoma mechanism of the six core genes was obtained by the comprehensive analysis of the component-target-pathway network, the relevant literature retrieval, and the screening of chemical components with minimum binding energy.

ESR1 is an estrogen receptor, which has a wide range of effects on the heterogeneous metabolism of human liver [29]. Liver is the target organ of sex hormone. The sex hormone level and the variants of sex hormone specific gene expression are supposed to be the main cause of liver disease [30]. It has been found that ESR1 is a direct downstream target of mir-393-3p, and mir-939-3p/ESR1 axis may be a potential new target for the treatment of liver cancer [31]. Verrier et al. found that ESR1 is one of the key host factors of the liver virus and a target for the inhibition of the liver virus [32]. Wang et al. also revealed that ESR1 is probably associated with HCC survival [33]. In this study, it was shown by target prediction and pathway enrichment that the bioactive compounds, glycolic acid hydrate, ginsenoside Rg1, and notoginsenoside R1, all act on the proteoglycans in cancer signal pathway and inhibit the progress of liver cancer by inhibiting the ESR1 transduction on the pathway.

Tyrosine kinase receptor 2 (ERBB2) is a member of the epidermal growth factor receptor (EGFR) family. Patients with high ERBB2 expression are prone to tumor metastasis and have a short survival period, which has become an ideal target of tumor immunobiotherapy and a hot molecule in the field of tumor therapy. It has been found that mir-296-5p inhibits EMT related metastasis of liver cancer through ERBB2 signaling pathway [34, 35]. Mir-375 upregulates cell proliferation and apoptosis by targeting ERBB2 and inhibits the growth of human hepatoma cells [36]. Yap directly regulates the transcription of ERBB2 and influences the proliferation of hepatocytes through ERBB2 signaling pathway [37]. Target prediction and pathway enrichment showed that the bioactive compounds, muscone and taurochenodeoxycholic acid, target ERBB2 through five signal pathways (pathways in cancer, prostate cancer, proteoglycans in cancer, focal adhesion, and bladder cancer) to inhibit the growth of liver cancer cells.

The insulin-like growth factor 1 receptor (IGF1R) is a transmembrane tyrosine protein located on chromosome 15q25-q26. In some malignancies, overexpression of IGF1R promotes tumor growth, invasion, and metastasis [38, 39]. Fan et al. believed that targeted IGF1R may be a promising therapeutic method for liver cancer [40]. Lin et al. believed that IGF1R and GRK2 are negatively correlated in liver cancer, and IGF1R may be a potential marker of poor prognosis in liver cancer [41]. Ye et al. found that the decrease of inhibitory effect on driving oncogene IGF1R and MAPK1 gene may trigger EMT and stem cell transformation of HCC [42]. According to target prediction and pathway enrichment, the bioactive compound stadium oxygen can act on the signal pathways of prostate cancer, pathways in cancer, proteoglycans in cancer, and focal adhesion and controls the progress of liver cancer by inhibiting the overexpression of IGF1R.

Androgen receptor (AR) may play a key role in influencing the progression of hepatocellular carcinoma at different stages [43, 44]. AR may be a marker for the response of patients with liver cancer to sorafenib [45]. AR signal transduction is involved in many aspects of the pathogenesis of liver cancer. The expression of AR-SV in liver cancer leads to the progress of liver cancer and the resistance to traditional AR antagonists. The successful therapy targeting AR-SVs is beneficial to liver cancer [46]. Target prediction and pathway enrichment revealed that 14 bioactive compounds (notoginsenoside R1, ginsenoside Re, ginsenoside Rg1, ginsenoside Rb1, ginsenoside Rd, chenodeoxycholic acid, cholic acid, glycodeoxycholic acid hydrate, deoxycholic acid, taurocholic acid, astragaloside A, ginsenoside Rd, sodium taurodeoxylate, and taurochenodeoxycholic acid) act on pathways in cancer signaling pathway and play a therapeutic role in liver cancer by regulating the expression of AR.

Notch signaling pathway is highly conserved and widely expressed in almost all tissues and organs, which plays a regulatory role in cell generation, development, differentiation, and proliferation. It has been reported that abnormal activation of Notch signal is related to the metastasis of hepatocellular carcinoma, and NOTCH1 can activate RNF187 promoter to promote the invasion and metastasis of hepatocellular carcinoma [47]. Inhibition of NOTCH1 signaling pathway may inhibit the development of HBx-induced hepatocellular carcinoma, and NOTCH1 may become a therapeutic target for hepatocellular carcinoma [48]. The bioactive compounds ginsenoside Rb1 and ginsenoside Rd inhibit the development of hepatocellular carcinoma by inhibiting the activation of NOTCH1 signal.

Albumin (ALB) is the most important protein in human plasma, which can maintain the nutrition and osmotic pressure and also reflect the synthesis function of the liver. It is found that the activity of ALB in patients with hepatitis, cirrhosis, and liver cancer is decreased significantly. ALB is considered to play a pivotal role in the inflammatory process and has been known as a prognostic indicator in several types of cancer [49]. Peng et al. found that ALB is a biomarker, which is related to the early diagnosis, metastasis, prognosis, or treatment of HCC [50]. The levels of ALB and GGT before operation are potential biomarkers to predict the prognosis of patients with liver cancer undergoing radical resection [51]. The evaluation of liver fibrosis degree is an important basis for the clinical diagnosis and treatment of HCC. Also, ALB is valuable while evaluating the liver fibrosis degree [52]. Chenodeoxycholic acid and cholic acid have good binding activity with ALB target genes, which can effectively regulate the activity of ALB, so as to improve the treatment status of patients with liver cancer.

Our KEGG pathway annotation analysis showed that some pathways were closely related to liver cancer [53]. The cyclin dependent kinase CDK2 is the target of tumor treatment [54]. The enrichment of 17 target genes in the pathways in cancer is primarily associated with liver cancer, such as Hsp90AB1e; the p53 signaling pathway may be an important molecular mechanism for the occurrence and development of liver cancer, which is consistent with the research results of Meng et al. [55] and Fan et al. [56]. The amplification of CCNE1 in this pathway is significantly related to the metastasis of liver cancer, which has been identified as an important target for tumor cells [57]. He et al. found that downregulation of CBL expression can inhibit the development of HCC [58]. In our study, the target genes of liver cancer are most abundant in metabolic pathways. Many studies have confirmed that this pathway is closely related to the occurrence and progress of HCC [59]. Sun et al. found that targeting specific metabolic pathways can prevent recurrence of liver cancer [60]. There are many target genes enriched in focal adhesion pathway, in which focal adhesion kinase and *β*-catenin collaboratively induce the production of HCC [61]. Also, many liver cancer target genes are enriched in the “proteoglycans in cancer” pathway. Proteoglycans are attractive biomarkers and therapeutic targets in hepatocellular carcinoma [62]. Purine metabolism pathway is also related to liver cancer. For example, the loss of dual specific tyrosine phosphorylation regulated kinase 3 can activate purine metabolism, which can promote the progress of liver cancer [63].

The degree of active ingredient indicates the number of correlations between the ingredient and target. According to the component-target-pathway network, sodium taurodeoxylate, taurochenodeoxycholic acid, glycocholic acid hydrate, chenodeoxycholic acid, ginsenoside Rd, muscone, cholic acid, and ginsenoside Rb1 are 8 bioactive compounds with high degree. They act on 6 core target genes and perform well in molecular docking. These provide a basis for further research on the bioactive compounds of antihepatoma.

## 5. Conclusions

PHT is mainly made from four compatible medicinal materials, which are *Calculus Bovis, Moschus, Rhizoma Notoginseng*, and *Snake Gall*. Its 16 chemical components act on different pathways by regulating different target proteins and collaboratively inhibit liver cancer, which shows the integrity and systematicness of Chinese traditional medicine prescriptions. ① Active small molecule ligands regulate liver cancer target gene receptors and inhibit the occurrence, development, and metastasis of liver cancer cells by interfering in tumor-related signaling pathways such as pathways in cancer, P53, proteoglycans in cancer, focal adhesion, metabolic and purine metabolism. ② Core target genes, ESR1, ERBB2, IGF1R, AR, NOTCH1,and ALB, are mainly involved in the transcription of RNA polymerase II promoters, steroid hormone mediated signaling pathway, protein autophosphorylation, positive regulation of apoptosis process, protein phosphorylation, transmembrane receptor protein tyrosine kinase signaling pathway, response to lipopolysaccharide, and other biological processes, which inhibits liver cancer. ③ Eight bioactive compounds (sodium taurodeoxylate, taurochenodeoxycholic acid, glycocholic acid hydrate, chenodeoxycholic acid, ginsenoside Rd, muscone, cholic acid, and ginsenoside Rb1) are effective to inhibit liver cancer. This study provides a basis for the molecular mechanism and biological process about how PHT inhibits liver cancer and lays a foundation for subsequent studies.

## Figures and Tables

**Table 1:** Information of active ingredients in PTH.

Traditional Chinese medicine (TCM)	No.	Active ingredient	Chemical Molecular Formula
*Rhizoma Notoginseng*	1	Notoginsenoside R1	C47H80O18
	2	Ginsenoside Re	C48H82O18
	3	Ginsenoside Rg1	C42H72O14
	4	Ginsenoside Rb1	C54H92O23
	5	Astragaloside A	C41H68O14
	6	Ginsenoside Rd	C48H82O18

*Calculus Bovis*	1	Taurine	C2H7NO3S
	2	Taurocholic acid	C26H45NO7S
	3	Glycocholic acid hydrate	C26H43NO6
	4	Cholic acid	C24H40O5
	5	Glycodeoxycholic acid hydrate	C26H43NO5
	6	Chenodeoxycholic acid	C24H40O4
	7	Deoxycholic acid	C24H40O4

*Moschus*	1	Muscone	C16H30O

*Snake galls*	1	Taurocholic acid	C26H45NO7S
	2	Glycocholic acid hydrate	C26H43NO6
	3	Sodium taurodeoxylate	C26H44NO6S
	4	Taurochenodeoxycholic acid	C26H45NO6S

**Table 2:** The degree value of 16 compounds in PTH.

No.	Chemical compound	Degree
1	Sodium taurodeoxylate	48
2	Taurochenodeoxycholic acid	46
3	Glycocholic acid hydrate	43
4	Glycodeoxycholic acid hydrate	41
5	Ginsenoside Re	40
6	Ginsenoside Rg1	40
7	Notoginsenoside R1	40
8	Chenodeoxycholic acid	39
9	Deoxycholic acid	38
10	Astragaloside A	34
11	Cholic acid	37
12	Ginsenoside Rb1	37
13	Ginsenoside Rd	37
14	Muscone	37
15	Taurocholic acid	34
16	Taurine	0

**Table 3:** Molecular docking of core genes and active components.

Core gene	Active ingredient	Combined energy
ESR1	Glycocholic acid hydrate	−6.4
AR	Chenodeoxycholic acid	−7.3
ALB	Chenodeoxycholic acid	−9.5
NOTCH1	Ginsenoside Rb1	−7.4
ERBB2	Taurochenodeoxycholic acid	−7.4
IGF1R	Sodium taurodeoxylate	−7.1

## Data Availability

The datasets generated for this study can be found at NCBI using accession number GSE76427 (https://www.ncbi.nlm.nih.gov/geo/query/acc.cgi?acc=GSE76427).
